# Glucocorticoids Improve in Vitro Mouse Oocyte Competence by Mimicking the Physiological Pre‐Ovulatory Environment

**DOI:** 10.1002/rmb2.70046

**Published:** 2026-04-02

**Authors:** Shahrina Akter, Takahiro Yamanaka, Asako Okamoto, Md Faizul Hossain Miraz, Masayuki Shimada

**Affiliations:** ^1^ Graduate School of Integrated Sciences for Life Hiroshima University Higashi‐Hiroshima Hiroshima Japan; ^2^ Bangladesh Livestock Research Institute Dhaka Bangladesh; ^3^ Department of Biological Sciences Asahikawa Medical University Asahikawa Japan; ^4^ Graduate School of Innovation and Practice for Smart Society Hiroshima University Higashi‐Hiroshima Hiroshima Japan

**Keywords:** corticosterone, cumulus cell, follicular fluid, glucocorticoid receptors, in vitro oocyte maturation techniques

## Abstract

**Purpose:**

Ovulation is a highly regulated inflammatory process that involves the initiation and timely resolution of the inflammation for efficient oocyte maturation. During this process, glucocorticoids accumulate in the pre‐ovulatory follicular environment as an anti‐inflammatory mediator. However, their temporal regulation, endogenous synthesis, and functional significance on oocyte maturation and subsequent developmental competence remain unclear.

**Methods:**

Corticosterone levels in reproductive tract fluid and serum, alongside the expression and localization of related markers, including glucocorticoid receptors (NR3C1), were analyzed in granulosa cells (GC) and cumulus cells (CC) during gonadotropin stimulation. The functional relevance of corticosterone was evaluated by inhibiting endogenous corticosterone synthesis using metyrapone (MET) and by supplementing exogenously during in vitro maturation (IVM) conditions.

**Results:**

Human chorionic gonadotropin (hCG) increased follicular corticosterone at 8 h and upregulated steroidogenic markers (*Cyp11a1*, *Cyp11b1*, and *Cyp21a1*) while downregulating the inactivating enzyme *Hsd11b2*. MET significantly inhibited FSH‐mediated endogenous corticosterone synthesis, resulting in poor IVM outcomes, and supplementation of exogenous corticosterone enhanced the IVM outcome, mitigated the MET effect, and maintained inflammatory balance.

**Conclusions:**

Gonadotropin induces a transient surge of ovarian corticosterone that supports in vivo maturation. Supplementing physiological levels of corticosterone during in vitro maturation (IVM) promotes oocyte maturation probably through cumulus cells.

## 1 Introduction

Ovulation is a complex biological event triggered by the surge of pre‐ovulatory luteinizing hormone (LH) that involves the initiation of a cascade of physiological events of follicular rupture and release of oocytes [1]. The landmark study of Espey in 1980 mentioned ovulation as an inflammatory response characterized by vascular changes, leukocyte infiltration, and tissue remodeling [2]. This response involves prostaglandin production, which has long been considered a key mediator of ovulation [3]. Prostaglandins synthesized by the induction of prostaglandin‐endoperoxide synthase 2 (*Ptgs2*) are critically important for the rupture of ovarian follicles and expansion of cumulus oocyte complexes [3, 4].

In addition to prostaglandins, LH surge causes granulosa cells to quickly produce an inflammatory cytokine profile. The release of these cytokines is regulated by a complex mechanism involving synaptosomal‐associated protein 25 (SNAP25), a component of the SNARE complex, which is necessary for the timely delivery of the inflammatory mediators to the follicular environment [5]. Additionally, an innate immune‐like signaling cascade is triggered when endogenous ligands within the follicle activate Toll‐like receptors (TLR2 and TLR4) [6, 7]. This pathway is critical for the release of particular cytokines, such as interleukin‐6 (IL‐6), which actively promotes cumulus expansion and results in the acquisition of oocyte developmental competence [8].

Resolution of this inflammation is an active process that restores tissue homeostasis [9]. In general, resolving this inflammation is a highly active and coordinated process that involves a transition from pro‐inflammatory signaling to the synthesis of specific pro‐resolving mediators (SPMs) that facilitate the repair of tissues [10, 11]. The primary endogenous coordinators of this resolution stage are glucocorticoids. The glucocorticoid receptor (GR; NR3C1), a ligand‐activated transcription factor, mediates their activity [12]. Following binding, glucocorticoids generally inhibit pro‐inflammatory transcription factors like NF‐κB and AP‐1 while simultaneously inducing the activation of anti‐inflammatory genes such as *AnxA1* and *Dusp1* [13, 14, 15]. However, several studies have also demonstrated that GR exerts biphasic effects on inflammatory responses, which enables selective activation of pro‐ and anti‐inflammatory mediators while also restraining excessive inflammation [16, 17]. Additionally, NF‐κB has been reported to be required for *Ptgs2* expression in granulosa cells [18]. In contrast, NF‐κB activity can be negatively regulated by the progesterone‐PGR axis [18]. However, this conclusion was based on experiments in which PGR activity was inhibited using RU486 [19, 20]. Because RU486 also acts as an antagonist of the glucocorticoid receptor [21], it is possible that a glucocorticoid‐mediated negative regulatory pathway for NF‐κB exists in granulosa cells as well. AP‐1 has been shown to regulate the expression of factors involved in progesterone production, and its activation following ovulatory stimulation has been reported [22]. AP‐1 is thought to enhance transcriptional activity through ERK1/2 signaling [23], and it is well known that ERK1/2 are essential for the ovulation process [24]; however, whether a mechanism exists to restrain excessive AP‐1 activity during the ovulatory process in granulosa cells remains unclear.

The time of glucocorticoid activation is an important yet little‐known feature of ovarian glucocorticoid biology. During follicular development, glucocorticoids limit follicle growth and can induce follicular atresia and apoptosis [25]. Conversely, during the pre‐ovulatory phase, glucocorticoids play a beneficial role by facilitating oocyte maturation and tissue remodeling following the LH surge [26]. These opposing effects suggest that ovarian glucocorticoid signaling must be tightly timed; however, when and where local production occurs, and how it relates to IVM conditions, remain unclear. This work investigated the timing of glucocorticoid synthesis and signaling during gonadotropin‐induced ovulation using a mouse superovulation model. We analyzed glucocorticoid concentrations in follicular fluid and circulation, as well as the expression and localization of glucocorticoid‐associated enzymes and receptors in granulosa cells and cumulus cells at specified pre‐ovulatory time points. Concurrently, we investigated the functional effects of glucocorticoids during in vitro maturation, emphasizing the impact of inhibiting endogenous synthesis and supplementing exogenously on oocyte maturation and developmental competence. This integrated study clarifies the timing and origin of synthesis and highlights a potential role of glucocorticoids in modulating in vivo and in vitro endocrine environment crucial for optimum oocyte maturation and developmental competence.

## 2 Materials and Methods

### 2.1 Chemicals and Reagents

Equine chorionic gonadotropin (eCG) and human chorionic gonadotropin (hCG) were purchased from Asuka Animal Health (Tokyo, Japan). Fetal bovine serum (FBS) was obtained from Thermo Fisher Scientific Inc. (10438–018, Waltham, MA, USA), and follicle‐stimulating hormone from Sigma‐Aldrich (FSH; F4021, St. Louis, MO, USA). We purchased Oligo (dT) primer (3805, Takara Bio Inc., Shiga, Japan) and Avian Myeloblastosis virus (AMV) reverse transcriptase (M5101, Promega, Madison, WI, USA). All other general chemicals and reagents used in this research were purchased from either Sigma‐Aldrich (St. Louis, MO, USA), Nacalai Tesque (Osaka, Japan), or FUJIFILM Wako Pure Chemical Corporation (Osaka, Japan). Antibody information used in this study is shown in Table 1.

**TABLE 1:** List of antibodies used for immunostaining and western blot analysis.

Target protein	Host	Cat. No.	Company	Application & Dilution used		
				WB	IF	IHC
CYP11B1	Rabbit	Bs‐3898R	Bioss Antibodies	1:300	1:400	
GR; NR3C1	Rabbit	42394	Cell Signaling Technology	1:1000		1:200
β‐Actin	Mouse	A5316	Sigma‐Aldrich	1:10000		
Rabbit IgG HRP‐linked antibody	Goat	7074S	Cell Signaling Technology	1:4000		
Mouse IgG HRP‐linked antibody	Horse	7076S	Cell Signaling Technology	1:4000		
Cy3‐conjugated anti‐rabbit IgG	Sheep	C2306	Sigma‐Aldrich		1:200	

### 2.2 Animal Management

All procedures required for conducting research with animals were properly maintained in accordance with institutional guidelines and approved by the Animal Care and Use Committee of Hiroshima University, Hiroshima, Japan (Approval No. C23‐36). For this study, immature C57BL/6NJ female mice (3 weeks) and Crl: CD1 (ICR) adult male mice (8–10 weeks) were purchased from Jackson Laboratory Japan (Kanagawa, Japan). Generally, immature female mice were injected intraperitoneally with 4 IU of eCG, followed 48 h later by 5 IU hCG for different time point sample collection. All mice were kept under controlled environmental conditions with a 12 h/12 h light/dark cycle, a 20°C–26°C temperature, and 40%–60% humidity with ad libitum access to food (MF; Oriental Yeast Co. Ltd., Tokyo, Japan) and water (Normal tap water).

### 2.3 Time‐Dependent Corticosterone Measurement by ELISA

Ovaries, oviducts, and blood samples were collected 48 h after eCG injection and at 4, 8, and 16 h after hCG administration. Follicular fluid was collected by puncturing visible follicles. Oviductal fluid was collected by gently flushing each oviduct with 500 μL PBS using a 26G needle. Samples were centrifuged at 800 × g for 5 min, and supernatants were collected as follicular fluid and oviduct fluid. For serum, blood samples were allowed to clot at room temperature for 15–20 min, followed by centrifugation at 1,800–2,000 × g for 10–15min. Corticosterone concentrations in the collected samples were quantified using the Corticosterone Enzyme Immunoassay Kit (K014‐H1; Arbor Assays, Ann Arbor, MI, USA) according to the manufacturer's protocol.

Serum samples were treated with a dissociation procedure before ELISA to quantify total corticosterone. Follicular/oviductal samples were assayed without dissociation; therefore, values reflect corticosterone measured under non‐dissociated conditions. Comparisons were performed within each sample type, and values across matrices were not directly compared. The colorimetric signal was measured using a microplate reader (Varioskan Flash, Thermo Fisher Scientific) at 450 nm wavelength, and final corticosterone concentrations were calculated based on the standard curve analysis.

**FIGURE 1:**
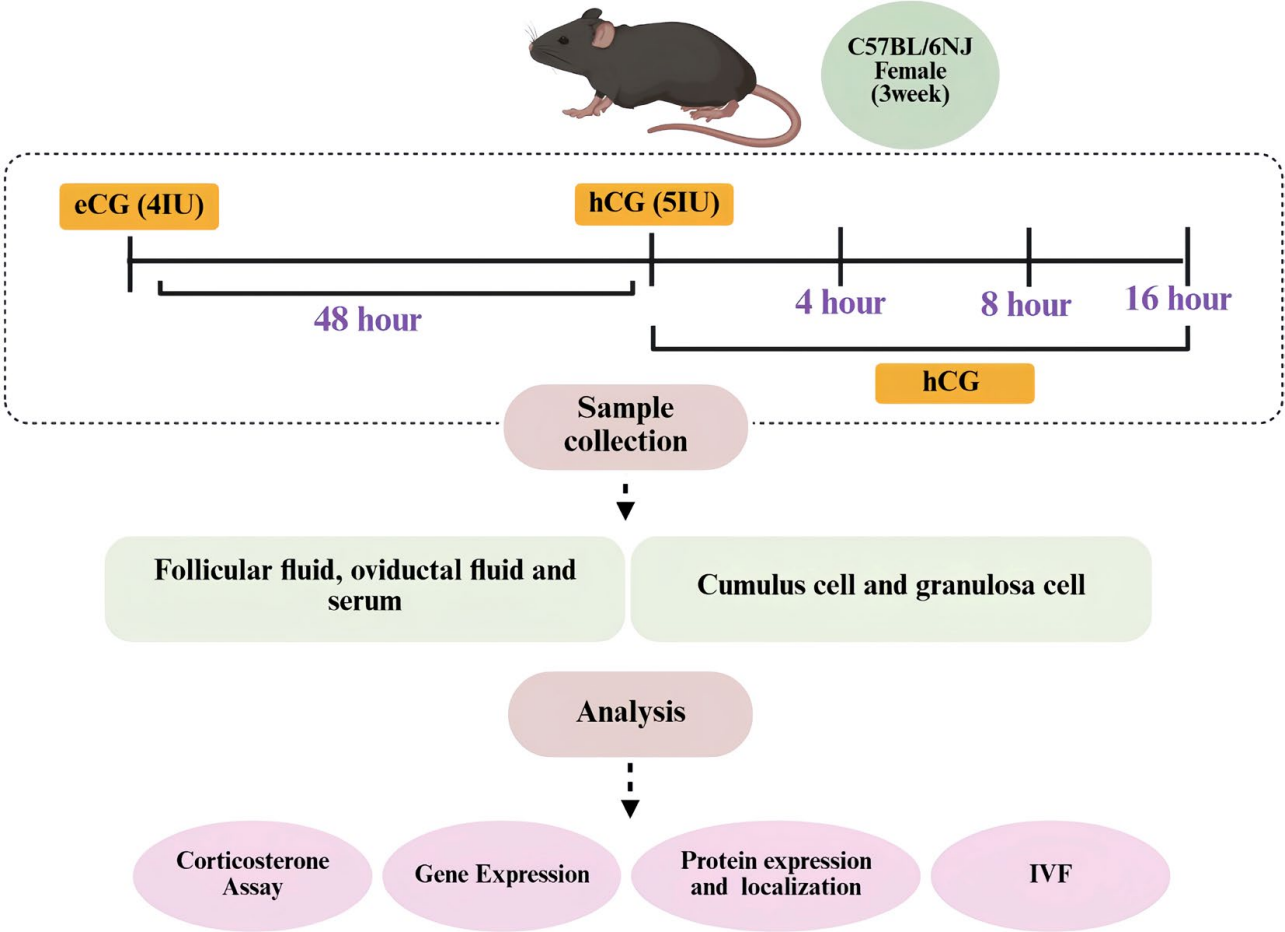
Summary of experimental design. This study was designed to investigate the effects of glucocorticoids in both in vivo and in vitro conditions. We first examined peri‐ovulatory changes in ovarian corticosterone and related signaling in vivo (eCG 48 h; hCG 4, 8, and 16 h). Corticosterone levels (ELISA), gene expression (qRT‐PCR), protein localization (IF/IHC), and protein abundance (western blot) were assessed in follicular/oviductal fluids, serum, cumulus cells, and granulosa cells. The role of endogenous corticosterone during in vitro maturation (IVM) was examined using a corticosterone synthesis inhibitor. Subsequently, the effects of exogenous corticosterone on IVM conditions were evaluated including their capacity to reverse the inhibitor‐induced effects and inflammatory responses. This figure was created with BioRender.com.

Protein concentrations in follicular and oviductal fluids were determined using the DC Protein Assay Reagent Kit (50000119, Bio‐Rad Laboratories, Hercules, CA, USA), according to the manufacturer's protocol. Briefly, sample aliquots were mixed with the reagent solution and incubated at room temperature, and absorbance was measured at 750 nm using a microplate reader. The detailed experimental layout is presented in Figure 1.

### 2.4 Quantitative Real‐Time PCR Analysis

Ovaries were collected at different time points (eCG48 h, hCG‐4, 8, 16 h) of gonadotropin stimulation. Cumulus cells were collected from the cumulus‐oocyte complex. After cumulus cell removal, granulosa cells were obtained from the remaining follicular tissue by centrifugation (600 × g, 5 min). Total RNA was extracted by RNeasy Mini Kit (74106, Qiagen, Venlo, Netherlands) following the manufacturer's protocol. RNA concentration was measured by a NanoDrop one microvolume spectrophotometer (Thermo Fisher Scientific, Waltham, MA, USA), and reverse transcription was performed according to a previous report [27, 28, 29]. Briefly, total RNA from granulosa cells and cumulus cells was mixed with 500 ng of Oligo (dT) primer and 0.25 U of AMV reverse transcriptase, followed by incubation at 42°C for 75 min and enzyme inactivation at 95°C for 5 min.

Quantitative real‐time PCR (qRT‐PCR) was performed with Power SYBR Green Master Mix in 15 μL reactions. Cycling conditions were 95°C for 2 min, then 40 cycles of 95°C for 3 s and 58°C–61°C (primer‐dependent) for 45 s (QuantStudio 3, Applied Biosystems, Waltham, MA, USA).

Primer specificity was confirmed by melt‐curve analysis and agarose gel electrophoresis. Only primer sets with single peaks and correct amplicon sizes were used. This study included testing each primer pair using known template cDNA to confirm successful amplification by analyzing the dissociation curve at the end of the qRT‐PCR run, including verification of the expected band sizes by agarose gel electrophoresis of PCR products. Only primer sets showing desired amplification and correct product sizes were used for further qRT‐PCR analysis, and all gene expression data were normalized by using the housekeeping gene *Rpl19*. Primer pairs used in this experiment are listed in Table 2.

**TABLE 2:** List of primer sequences used for qRT‐PCR.

Gene	Primer sequences	Product size (bp)	Annealing temperature (°C)	Accession number
***Cyp11a1***	F: 5′‐GGGAGACATGGCCAAGATGG‐3′ R: 5′‐CAGCCAAAGCCCAAGTACCG‐3′	279	60	*NM_019779.4*
***Cyp21a1***	F: 5′‐GCTTCATTTCCGTGAAGGGC‐3′ R: 5′‐GGCATTGATATGGCGCTGTC‐3′	118	60	*NM_009995.2*
***Cyp11b1***	F: 5′‐GTCAGATTCACTTGGGGGCA‐3′ R: 5′‐GGACATGCCCTCCCTGTATG‐3′	117	60	*NM_001033229.4*
***Hsd11b2***	F: 5′‐GCGTGACCTCTGTTCTCCTC‐3′ R: 5′‐GTCAGCTCAAGTGCACCAAA‐3′	228	58	*NM_008289.2*
***Rpl19***	F: 5′‐GGCATAGGGAAGAGGAAGG‐3′ R: 5′‐GGATGTGGTCCATGAGGATGC‐3′	199	60	*NM_009078.2*

### 2.5 Immunohistological Analysis

Ovaries were fixed in 4% paraformaldehyde overnight at 4°C, dehydrated in graded ethanol, cleared, and embedded in paraffin. Sections (5 μm) were cut and mounted on slides. Deparaffinization was performed using Fast Solve (306–501‐1, Pharma Co. Ltd., Tokyo, Japan), followed by graded ethanol rinses. To block the endogenous peroxidase activity, slides were incubated with 3% hydrogen peroxide in methanol for 10 min at room temperature and permeabilized with 0.3% (v/v) Triton X‐100/PBS (−) for 15 min. Antigen retrieval was performed by boiling the slides in 10 mM citric acid buffer at 98°C for 15 min, followed by cooling to room temperature and washing 2–3 times with PBS (−) for 2 min each.

For Immunohistochemistry sections were blocked with 2.5% Normal Horse serum for 20 min to block nonspecific sites, then incubated overnight with primary antibodies, Anti‐glucocorticoid receptor (GR; NR3C1) (1:200; 42394; Cell Signaling Technology). Positive signals were visualized by using DAB (3,3′‐Diaminobenzidine, 040–27001, Fujifilm Wako, Osaka, Japan) according to the ImmPRESS HRP Horse Anti‐Rabbit IgG PLUS Polymer Kit, Peroxidase (MP‐7801, Vector Laboratories) protocol, followed by nuclear counterstaining with hematoxylin (Sakura Finetek, Tokyo, Japan).

For Immunofluorescence blocking was performed with Normal Goat Serum (S‐1000, Vector Laboratories, Newark, CA, USA) in PBS (−) (PBS: Goat serum; 1000 μL: 15 μL) for 1 h at room temperature and incubated overnight with Primary antibody Anti‐CYP11B1 (1:400; bs‐3898R; Bioss Antibodies) at 4°C. After primary antibody incubation, slides were washed with 0.3% (v/v) Triton X‐100 in PBS (−), and incubated with goat anti‐rabbit IgG secondary antibody (1:200, C‐2306, Sigma‐Aldrich). After incubation with secondary antibody (2 h, RT, dark), sections were washed and counterstained with DAPI (H‐1200, VECTESHIELD Mounting Medium with DAPI, Vector Laboratories). All images were taken using an APX100 Digital Imaging System (EVIDENT, Tokyo, Japan).

### 2.6 Western Blot Analysis

For western blot analysis, cumulus cells and granulosa cells were collected at a similar time point of gonadotropin stimulation. Protein was extracted from cumulus cells and granulosa cells by homogenizing in cell lysis buffer (04719964001, cOmplete Lysis‐M EDTA‐free, Roche, and 04906837001, PhosSTOP EASY pack, Roche) and diluted with the same volume of SDS buffer, followed by boiling at 100°C for 10 min to denature the protein. Protein concentrations were measured using the BIO‐RAD DC Protein Assay Kit according to the manufacturer's protocol. 10 μg of protein from each sample (10 μL) were separated by SDS‐PAGE using a 10% polyacrylamide gel and subsequently transferred onto a Polyvinylidene fluoride (PVDF) membrane (10600069, Cytiva, Tokyo, Japan). Membranes were blocked in TBST containing 5% skim milk for 2 h at RT, then incubated overnight at 4°C with primary antibodies (CYP11B1, 1:300; NR3C1, 1:1000; β‐actin, 1:10000). The next day, the PVDF membranes were washed with TBS‐T and incubated for 2 h with HRP‐conjugated secondary antibodies specific to mouse and rabbit IgG (1:4000 dilution; 7076S and 7074S; Cell Signaling Technology). Band detection was carried out using an enhanced chemiluminescence (ECL) system (RPN2232, Cytiva) and visualized with the ChemiDoc MP Imaging System (Bio‐Rad Laboratories). Band intensity was measured by using the ImageJ software (National Institutes of Health, Bethesda, MD, USA). The full‐length blotting images are shown in Figure S1.

### 2.7 Effects of Metyrapone (MET) on in Vitro Culture and in Vitro Fertilization (IVF)

Ovaries were collected from immature mice 48 h after eCG injection. Cumulus‐oocyte complexes (COCs) were released by puncturing the follicle with a needle and collected by a glass pipette. Twenty COCs were cultured in 100 μL droplets of low‐glucose DMEM (041–29975, Fujifilm, Japan) supplemented with 1% FBS and 2 mM hypoxanthine (H9377, Sigma‐Aldrich, St. Louis, MO, USA) under paraffin oil. Maturation medium was supplemented with FSH (Final concentration: 0.1 IU/mL) and different doses of MET (Final concentration 0, 5, 10, 20, and 100 μM) (sc‐200597, Santa Cruz Biotechnology, CA, USA) and finally incubated for 16 h at 37°C with 5% CO₂. Morphology and cumulus expansion were visualized and imaged by using a stereo microscope (SZ61, Olympus, Tokyo, Japan) equipped with a digital camera at maximum zoom (4.5×). COC diameter was quantified by using ImageJ software (National Institutes of Health, Bethesda, MD, USA).

After IVM, COCs were washed two to three times in HTF. Spermatozoa were collected from the cauda epididymis of ICR mice into 400 μL HTF and capacitated for 1.5 h at 37°C. Following capacitation, 5–6 μL of sperm suspension was added to the fertilization droplet to achieve a final concentration of 2 × 10^5^ sperm/mL and incubated for 6 h under the same conditions used for IVM. After 6 h, zygotes were washed 4–5 times in KSOM to remove sperm and cumulus cells. Pronuclei and polar bodies were assessed by phase‐contrast microscopy. Embryos were then cultured in 50 μL KSOM (MR‐106‐D, Sigma‐Aldrich) droplets for 5 days.

Fertilization was assessed as the 2‐cell rate at 24 h post‐insemination. Blastocyst formation was assessed on day 5 among cleaved embryos. MET stocks were prepared by dissolving the powder in dimethylsulfoxide (DMSO; 037–24053, Sigma‐Aldrich, St. Louis, MO, USA) with a concentration of 100 mM and stored at −30°C. The final treatment concentrations were prepared by serial dilution with the maturation medium at the proper ratio. The final concentration of DMSO in the medium was ≤ 0.1% (v/v) and this concentration did not affect the cumulus cell function during meiosis process [30].

### 2.8 Quantification of Endogenous Corticosterone Levels in Culture Media

For confirmation of endogenous corticosterone synthesis from COCs induced by FSH and the actual inhibitory effect of MET, droplets were collected after 16 h of COCs culture, which was supplemented with FSH (0.1 IU/mL) along with different doses of MET (0, 5, 10, 20, and 100 μM). Following centrifugation at 10,000 × g for 20 min, the supernatants without dissociation were analyzed for corticosterone by ELISA as described in Section 2.3. However, the only modification was that corticosterone standards were prepared using the same culture medium used in the IVM conditions, instead of the assay buffer, for more accurate quantification, and also avoiding the matrix inconsistencies between standards and samples.

### 2.9 Exogenous Corticosterone Intervention Study: In Vitro Maturation and Subsequent Developmental Competence

Immature COCs were collected after eCG primed mouse and cultured for 16 h for maturation. Maturation medium was supplemented with FSH (0.1 IU/mL) and different doses of corticosterone. The following concentrations of corticosterone were used for treatment: 0, 1, 10, 100, and 1000 pg/mL (C2505, Sigma‐Aldrich, St. Louis, MO, USA) and incubated for 16 h at 37°C with 5% CO2. Morphology and cumulus expansion were visualized and imaged accordingly. IVF and in vitro culture were performed as previously described. Corticosterone was dissolved in DMSO (stock: 1 mg/mL). Stocks were stored at −30°C and diluted into medium immediately before use. The final concentration of DMSO in the medium < 0.1% (v/v) and this concentration did not affect the cumulus cell function during meiosis process [30].

### 2.10 Inhibitor Recovery Experiment

To evaluate whether exogenous corticosterone could reverse the inhibitory effects of MET, immature COCs were collected from eCG primed mice and cultured for 16 h, supplemented with FSH under four conditions: control, MET (20 μM), and a combination of MET (20 μM) and corticosterone (100 pg/mL). Oocyte maturation rate and subsequent developmental competence were evaluated accordingly.

### 2.11 The Gene Expression of Cytokine and Chemokine Family

To understand the role of corticosterone on the physiological pre‐ovulatory microenvironment, we evaluated the cytokine and chemokine expression profile during the in vivo and in vitro pre‐ovulatory phase. In vivo, COCs were collected at two points: eCG 48 h and hCG 8 h. The expression profile of selected cytokines and chemokines was analyzed by qRT‐PCR as described in Section 2.4.

For in vitro study, immature COCs were collected from eCG‐primed mouse ovaries. Total COCs were randomly divided into three groups: (1) Baseline control‐eCG 48 h (0 h), (2) 8 h IVM without corticosterone (0 pg/mL), and (3) 8 h IVM with 100 pg/mL corticosterone supplementation. For baseline control, COCs were collected just after eCG 48 h injection, and the other two group COCs were cultured with 0 pg/mL or 100 pg/mL corticosterone for 8 h. qRT‐PCR was performed similarly as described in Section 2.4. Primer pairs used in this experiment are listed in the Table S1.

### 2.12 Statistical Analysis

All data were collected from at least three biological replicates and are presented as mean ± standard error of the mean (SEM). Data normality and homogeneity of variance were tested by using the Shapiro–Wilk test and Bartlett's test before statistical analysis. Comparison between multiple groups was performed by one‐way or two‐way analysis of variance (ANOVA), as appropriate, followed by either Tukey's honest significant difference (HSD) test or Dunnett's test. GraphPad Prism (version 8.0.2) (GraphPad Software, San Diego, CA, USA) was used for all statistical analyses, and significance was set at *p* < 0.05.

## 3 Results

### 3.1 Corticosterone Level Increased in Follicular Fluid Following hCG Administration

**FIGURE 2:**
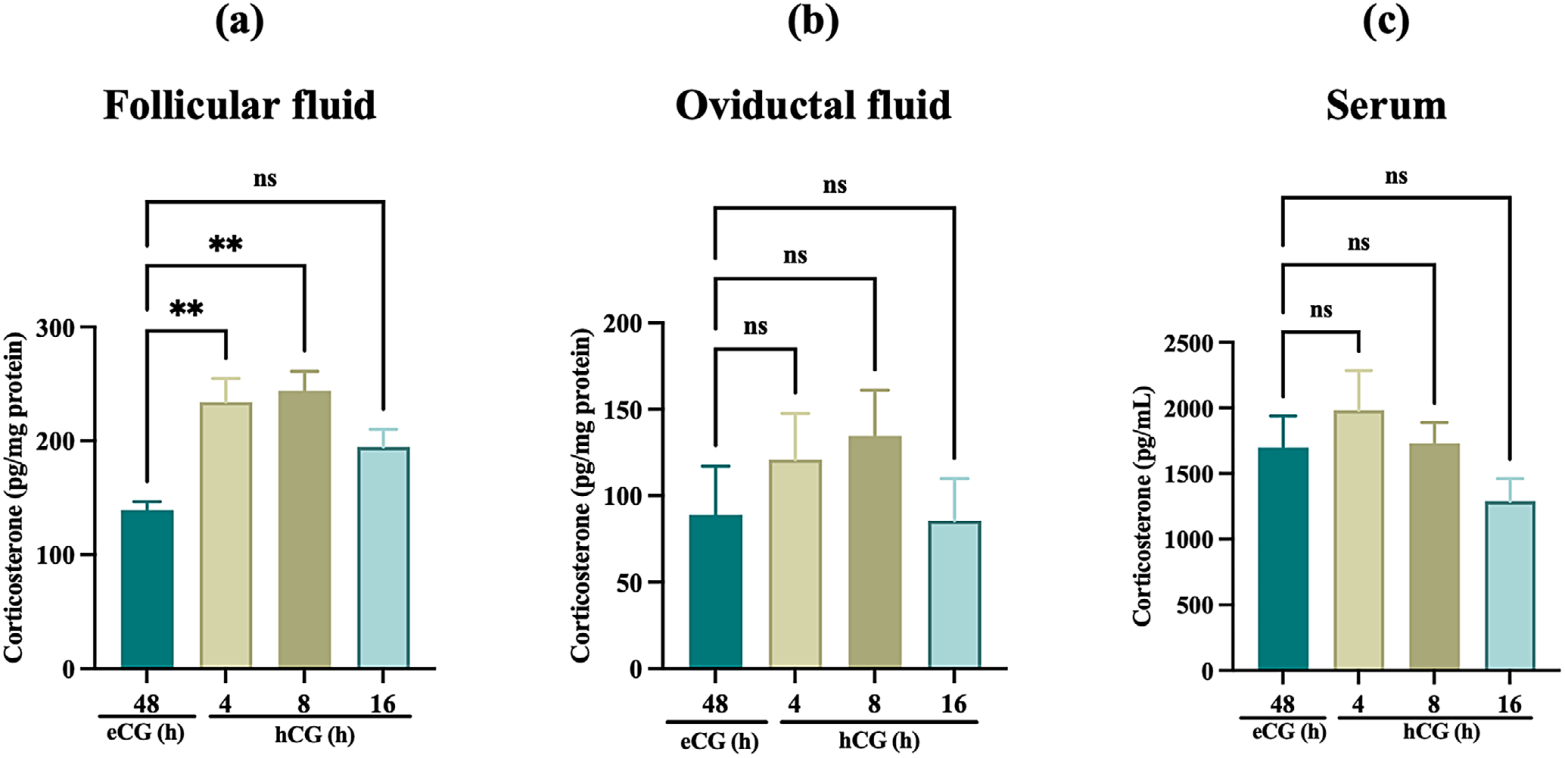
Corticosterone level in reproductive tract fluid and circulatory system at different ovulatory stage. A significant increase in corticosterone concentration in follicular fluid was observed after gonadotropin administration. (a–c) Corticosterone concentrations in follicular fluid (a) oviductal fluid (b) and serum (c) at eCG 48 h and hCG 4, 8, and 16 h. Follicular and oviductal fluid values were normalized to total protein. Data are mean ± SEM from ≥ 3 independent experiments. One‐way ANOVA with Dunnett's test vs. control (eCG 48 h). **p < 0.01. eCG, equine chorionic gonadotropin; h, hours; hCG, Human chorionic gonadotropin; ns, non‐significant; SEM, standard error of the mean (*p* > 0.05).

We measured corticosterone in follicular fluid, oviductal fluid, and serum by ELISA across the hCG time course. Corticosterone levels were significantly increased in follicular fluid following hCG stimulation (*p* < 0.05) (Figure 2a); however, no significant changes were observed in oviductal fluid and serum (*p* > 0.05) (Figure 2b,c), suggesting that corticosterone becomes selectively elevated within the follicular microenvironment following hCG stimulation, consistent with locally regulated glucocorticoid signaling.

### 3.2 Administration of hCG Up‐Regulated Corticosterone Synthesis Genes While Progressively Down‐Regulated Metabolizing Genes

**FIGURE 3:**
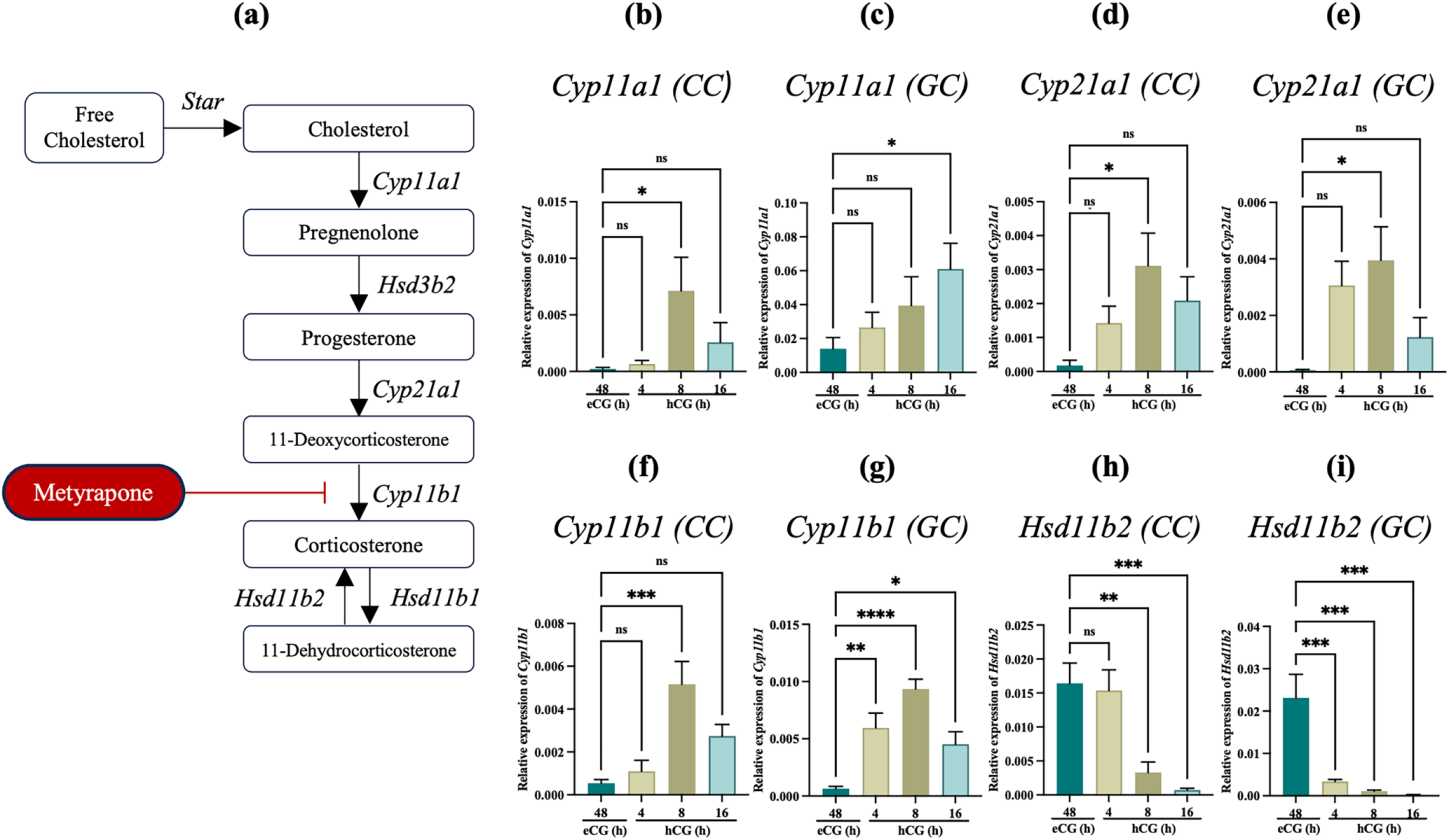
Expression of corticosterone synthesis enzymes in cumulus cells (CC) and granulosa cells (GC) at different ovulatory stages (a) Corticosterone hormone synthesis pathways in Rodents. (b–i) qRT‐PCR analysis of *Cyp11a1* (b, c) *Cyp21a1* (d, e) *Cyp11b1* (f, g) and *Hsd11b2* (h, i) in CCs and GCs at eCG 48 h and hCG 4, 8, and 16 h. Expression was normalized to *Rpl19*. Values are presented as the mean ± SEM of more than three independent experiments. Data were analyzed using one‐way ANOVA followed by Dunnett's multiple comparisons test, comparing each treatment group with the control (eCG 48 h). *p < 0.05, **p < 0.01, ***p < 0.001, ****p < 0.0001. CC, Cumulus cell; eCG, equine chorionic gonadotropin; GC, granulosa cell; h, hours; hCG, human chorionic gonadotropin; Metyrapone, inhibitor of CYP11B1 activity; ns, non‐significant SEM, standard error of the mean (*p* > 0.05).

Administration of hCG stimulated the corticosterone production in the ovarian environment, suggesting that ovarian cells, e.g., cumulus cells and granulosa cells, may contribute to the corticosterone synthesis (Figure 3a). Regulation of hCG‐mediated corticosterone synthesis and metabolism‐related gene expression in cumulus cells and granulosa cells was evaluated by qRT‐PCR. The expression of genes encoding enzymes to synthesize corticosterone (*Cyp11a1*, *Cyp21a1*, *Cyp11b1*) was significantly up‐regulated in both cumulus cells and granulosa cells after hCG stimulation (*p* < 0.05) (Figure 3b–g), whereas the gene encoding metabolized enzyme (*Hsd11b2*) responsible for inactivation of active corticosterone was significantly down‐regulated pattern in both cell types (*p* < 0.05) (Figure 3h,i). These results support increased corticosterone synthesis during the pre‐ovulatory phase, consistent with a transiently permissive environment for oocyte maturation.

### 3.3 Corticosterone Synthesizing Enzyme (CYP11B1) and Glucocorticoid Receptor (GR; NR3C1) Localized in Ovarian Cells, Which Were Stimulated Upon hCG Administration

**FIGURE 4:**
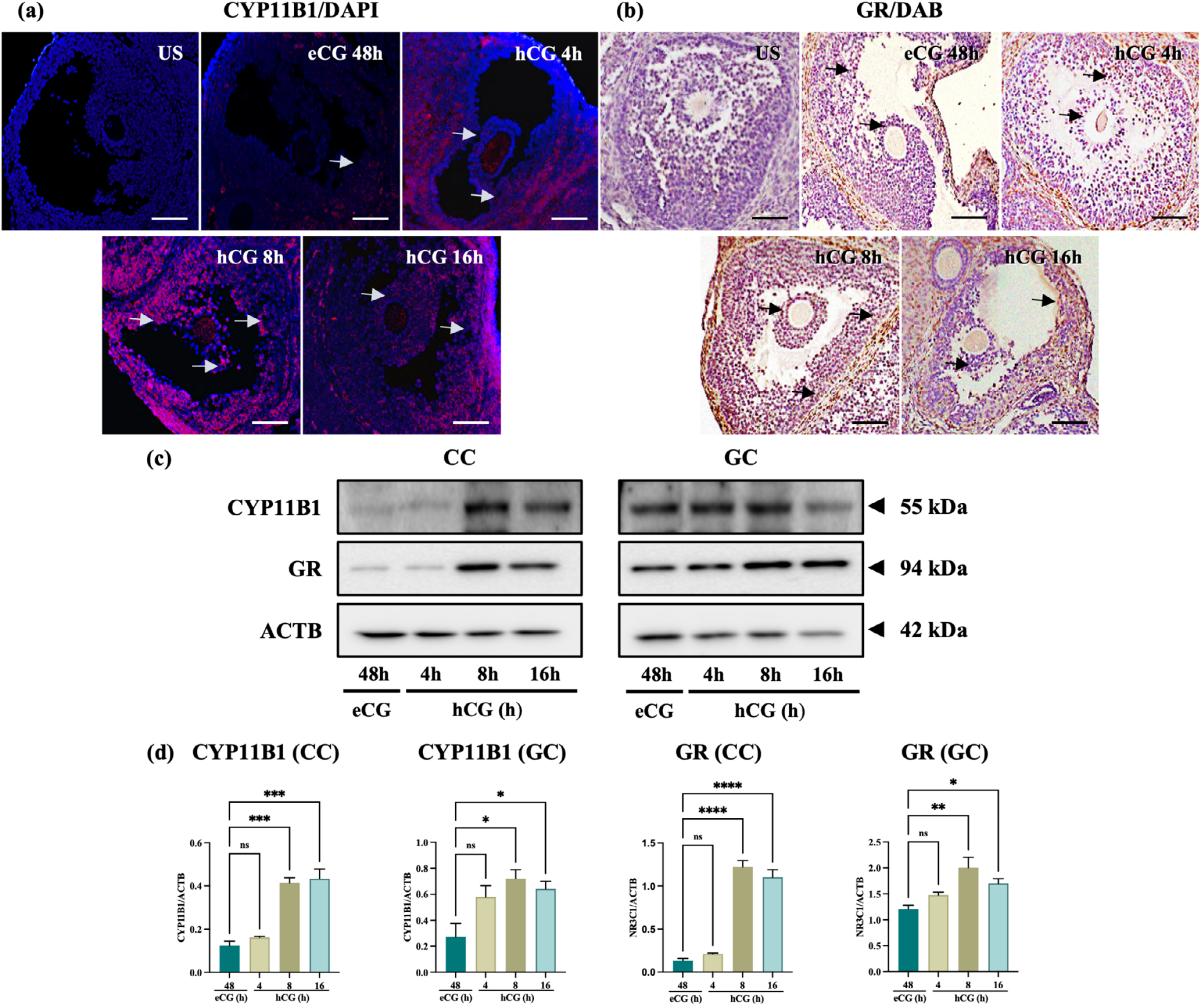
Localization and expression of CYP11B1 and glucocorticoid receptor (GR; NR3C1) in cumulus cell (CC) and granulosa cell (GC) at different ovulatory phases (a, b) Representative immunostaining showing CYP11B1 and GR in ovarian follicles at eCG 48 h and hCG 4, 8, and 16 h (scale bar, 50 μm). (c) Western blot analysis of CYP11B1 and GR in CCs and GCs. ACTB was used as a loading control. (d) Quantification of protein levels relative to ACTB; uncropped blots are shown in Figure S1. Analyzed values are presented as the mean±SEM of three independent experiments. Data were analyzed using one‐way ANOVA followed by Dunnett's multiple comparisons test, comparing each treatment group with the control (eCG 48 h). *p < 0.05, **p < 0.01, ***p < 0.001, ****p < 0.0001. CC, cumulus cell; eCG, equine chorionic gonadotropin; GC, granulosa cell; h, Hours; hCG, human chorionic gonadotropin; ns, non‐significant; SEM, standard error of the mean (*p* > 0.05).

Since the genes encoding corticosterone conversion enzyme were expressed in cumulus cells and granulosa cells, we focused on their localization and protein levels by immunohistological analysis and western blotting. Both CYP11B1 and GR were detected in cumulus cells and granulosa cells with an increasing expression following hCG administration (Figure 4a,b). Consistent with *Cyp11b1* mRNA expression data, CYP11B1 protein was poorly expressed prior to hCG but increased notably at 8 h after hCG administration (Figure 4a). Similarly, GR was detected in granulosa cells at all time points, whereas cumulus cells showed stronger nuclear GR signals after hCG (Figure 4b). Western blot also showed elevated expression of both CYP11B1 and GR after hCG injection in cumulus cells and granulosa cells (Figure 4c,d), consistent with our previous quantitative study. Both proteins showed significantly higher expression at 8 h post‐hCG (*p* < 0.05) (Figure 4d).

### 3.4 Endogenous Corticosterone Is Essential for Meiotic Progression and in Vitro Embryo Development

**FIGURE 5:**
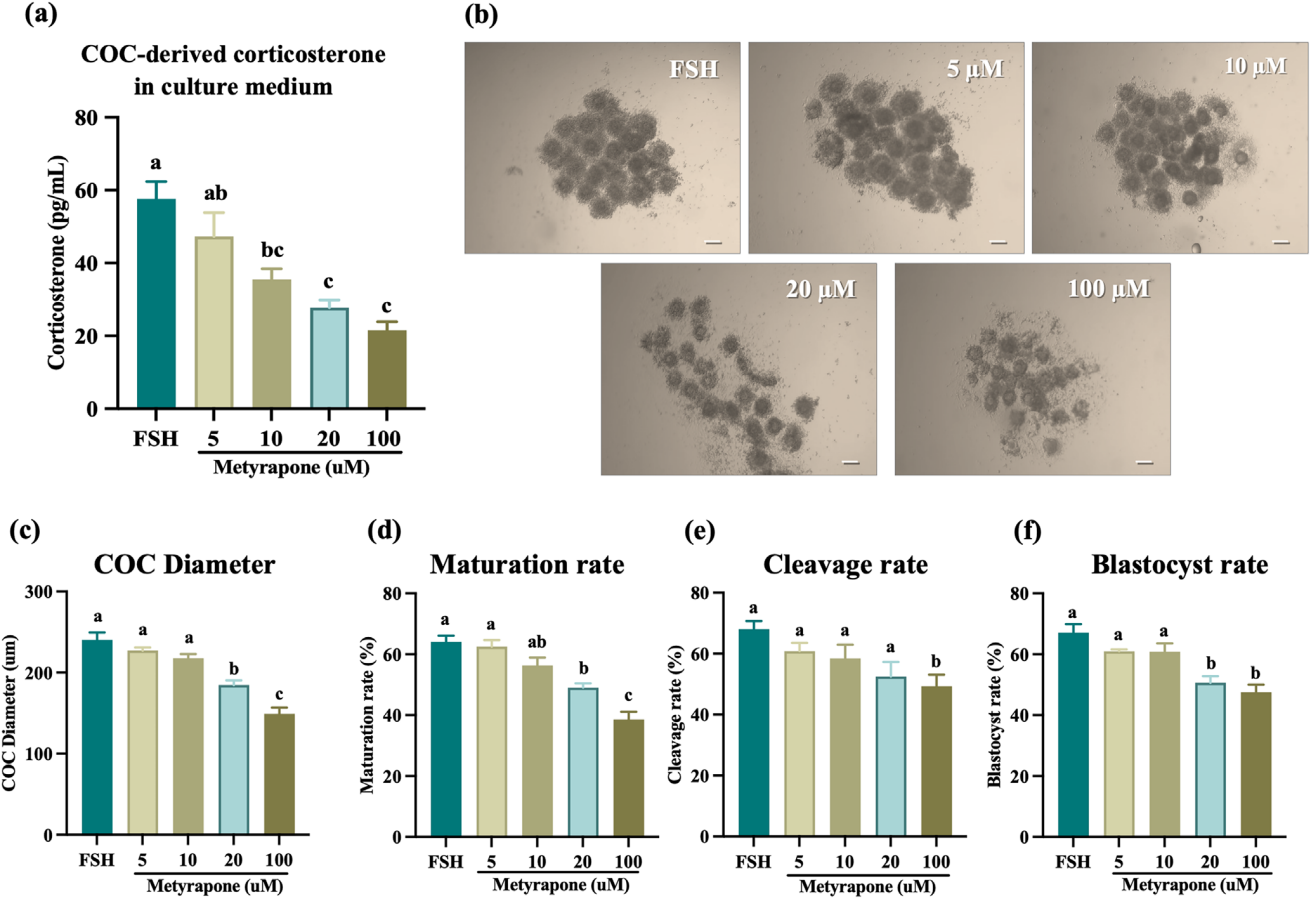
Effects of Metyrapone (MET) on endogenous corticosterone synthesis and in vitro oocyte competence. MET impairs endogenous corticosterone production and oocyte competence during IVM. (a) Cumulus oocyte complexes (COC)‐derived corticosterone in culture supernatants after 16 h of IVM with FSH ± MET. (b–f) Dose‐dependent effects of MET (0–100 μM) on COC morphology (b; scale bar, 200 μm), cumulus expansion (c) MII rate (d) 2‐cell rate (e) and blastocyst rate (f) Values are presented as the mean ± SEM of more than three independent experiments. Different lowercase letters indicate significant differences (*p* < 0.05). Data were analyzed using one‐way ANOVA followed by Tukey HSD post hoc test. COCs, cumulus oocyte complexes; eCG, equine chorionic gonadotropin; IVF, In vitro fertilization; MET, metyrapone; MII, metaphase‐II; SEM, standard error of the mean.

In vivo analysis showed that hCG induced the endogenous corticosterone production in the ovary. For confirmation of FSH‐mediated endogenous corticosterone synthesis from cumulus cells, corticosterone concentration in culture media was measured by ELISA, thereby verifying both FSH‐induced corticosterone production and the effective inhibition of CYP11B1 activity by MET. FSH supplementation induced endogenous corticosterone production in the culture medium; however, this concentration was significantly reduced with the addition of MET in a dose‐dependent manner (Figure 5a). The maximum inhibitory effects were observed at 20 μM of MET. MET also impaired cumulus expansion, oocyte maturation rate, and other developmental parameters in a dose‐dependent manner, with significant reductions observed at 20 μM doses (Figure 5b–f). These findings suggested that blocking endogenous corticosterone synthesis disrupts meiotic progression and embryo developmental potential.

### 3.5 Exogenous Corticosterone Supplementation Enhanced in Vitro Maturation Rate and Developmental Competence

**FIGURE 6:**
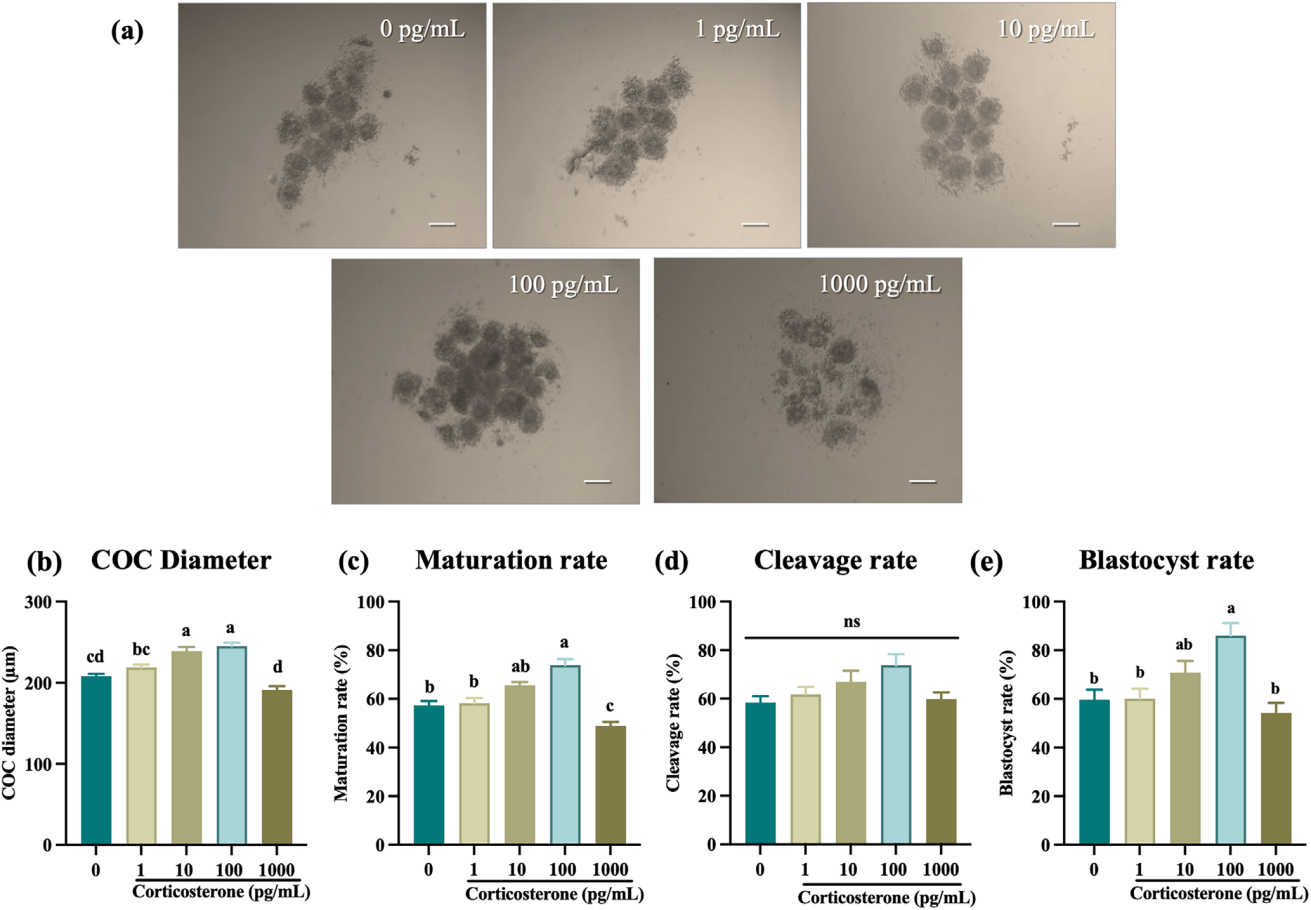
Effects of exogenous corticosterone on in vitro maturation and developmental competence. Exogenous corticosterone improves oocyte maturation and developmental competence in vitro. (a–e) Dose‐dependent effects of corticosterone (0, 1, 10, 100, 1000 pg/mL) during 16 h IVM on morphology (a; scale bar, 200 μm), COC diameter (b) MII rate (c) 2‐cell rate (d) and blastocyst rate (e) Values are presented as the mean ± SEM of more than three independent experiments. Different lowercase letters indicate significant differences (*p* < 0.05). Data were analyzed using one‐way ANOVA followed by Tukey HSD post hoc test. COCs, cumulus oocyte complexes; MII, metaphase‐II; pg/mL, picograms per milliliter; SEM, standard error of the mean.

Although follicular fluid corticosterone levels increase after hCG administration (Figure 2a), our gene and protein data suggest that both cumulus and granulosa cells express key components of the corticosterone synthesis and signaling machinery (Figures 3 and 4). This indicates that multiple follicular compartments may contribute to the local glucocorticoid levels. Therefore, we thought that not only endogenous corticosterone from cumulus cells of COCs but also added corticosterone showed even more positive effects in the culture system of COCs. To test this hypothesis, we supplemented exogenous corticosterone in vitro to mimic the in vivo environment, focusing on enhancing the oocyte maturation rate and developmental competence. Exogenous corticosterone supplementation significantly improved the IVM outcome. Corticosterone supplementation enhanced the oocyte quality by increasing the COCs' expansion and compactness (Figure 6a,b). The addition of 100 pg/mL corticosterone significantly enhanced the oocyte maturation rate and other developmental parameters while a higher dose (1,000 pg/mL) markedly reduced all these parameters (*p* < 0.05; Figure 6c–e). Thus, supplementation with 100 pg/mL exogenous corticosterone maintained a favorable in vitro microenvironment, thereby enhancing the oocyte competence.

### 3.6 Exogenous Corticosterone Restored MET‐Induced Suppression Under IVM Conditions

**FIGURE 7:**
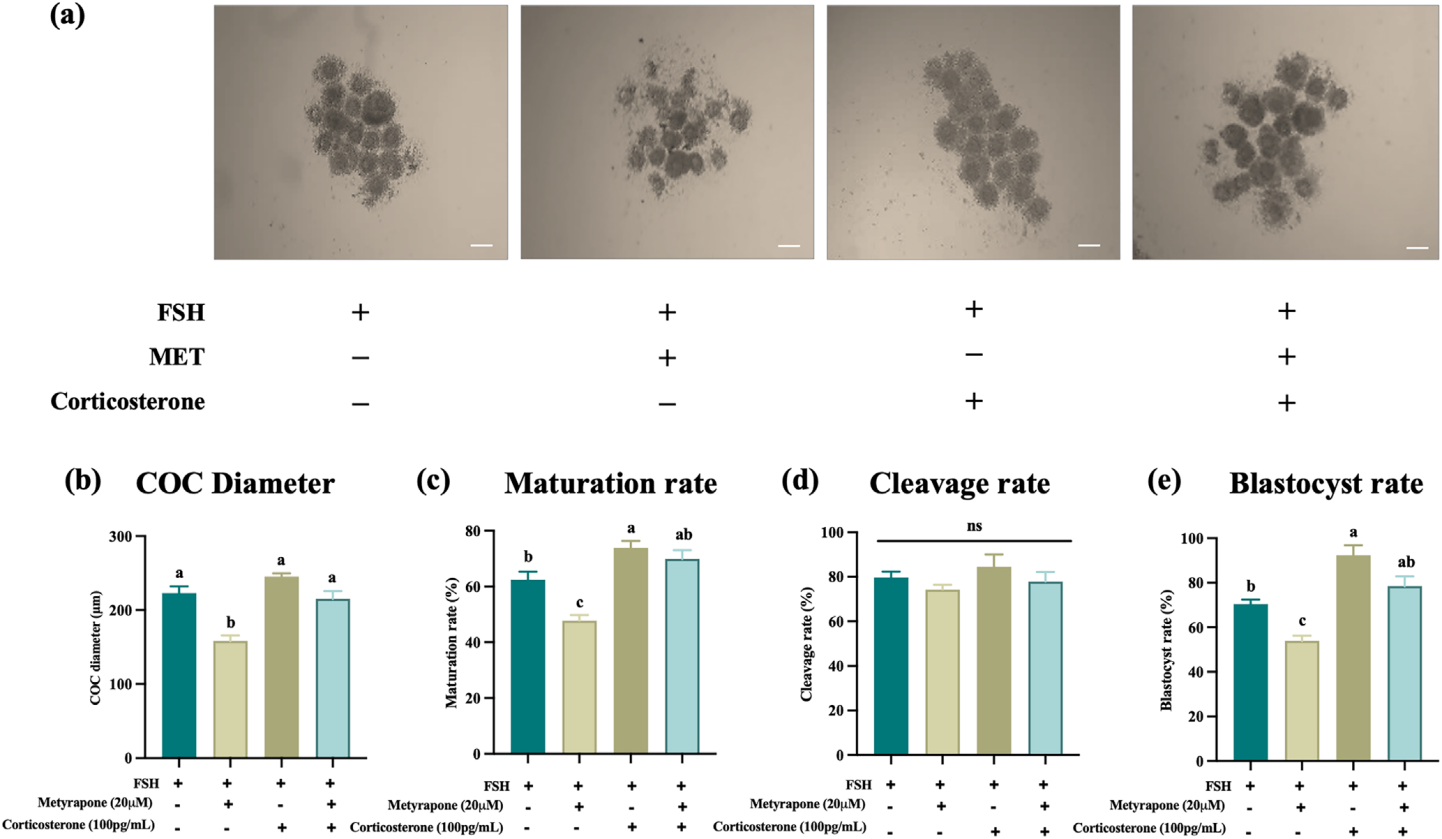
Recovery effects of exogenous corticosterone on Metyrapone (MET) inhibition. Corticosterone rescues MET‐induced impairment of oocyte competence. (a–e) Morphology (a) COC diameter (b) MII rate (c) 2‐cell rate (d) and blastocyst rate (e) in COCs cultured with FSH alone, FSH + MET (20 μM), FSH + corticosterone (100 pg/mL), or FSH + MET + corticosterone. Two‐way ANOVA (MET × corticosterone) with Tukey's post hoc test. Two‐way ANOVA revealed significant main effects of MET and corticosterone, with no interaction (*p* > 0.05; b, c, e). Values are presented as the mean±SEM of more than three independent experiments. Different lowercase letters indicate significant differences (*p* < 0.05). COCs, cumulus oocyte complexes; IVF, In vitro fertilization; MET, metyrapone; MII, metaphase‐II; SEM, standard error of the mean.

For further understanding and elucidating the role of corticosterone, MET was added with FSH containing culture medium to inhibit the endogenous corticosterone synthesis. Exogenous corticosterone (100 pg/mL) was supplemented to rescue the inhibitory effects of MET. Treatment with MET (20 μM) significantly suppressed maturation rate, COCs diameter, and blastocyst rate (*p* < 0.05). However, exogenous corticosterone (100 pg/mL) significantly rescued these MET‐induced impairments (*p* < 0.05; Figure 7a–e).

### 3.7 Exogenous Corticosterone Mimics Pre‐Ovulatory Conditions by Inducing Cytokine and Chemokine Expression in Vitro

Our in vivo time‐course analysis identified 8 h post‐hCG as a critical time window of maximal endogenous corticosterone activation. This phase of the ovulatory cycle is generally maintained by several key pro‐ and anti‐inflammatory cytokines and chemokines. Consistent with this, our in vivo analysis demonstrated that during the preovulatory phase, selected cytokines and chemokines (*Il6, Il7, Il‐1β, Cxcl1, and Il10*) were significantly up‐regulated (*p* < 0.05, Figure S2a).

We further investigated whether exogenous corticosterone supplementation during in vitro culture conditions could mimic this physiological inflammatory profile. Cumulus cells were collected at 8 h post‐IVM with (100 pg/mL) or without (0 pg/mL) corticosterone, and the expression level was compared with baseline control (eCG 48 h). Exogenous corticosterone significantly up‐regulated all selected cytokines and chemokines (*Il6, Il7, Il‐1β, Cxcl1, and Il10*) (*p* < 0.05, Figure S2b).

**FIGURE 8:**
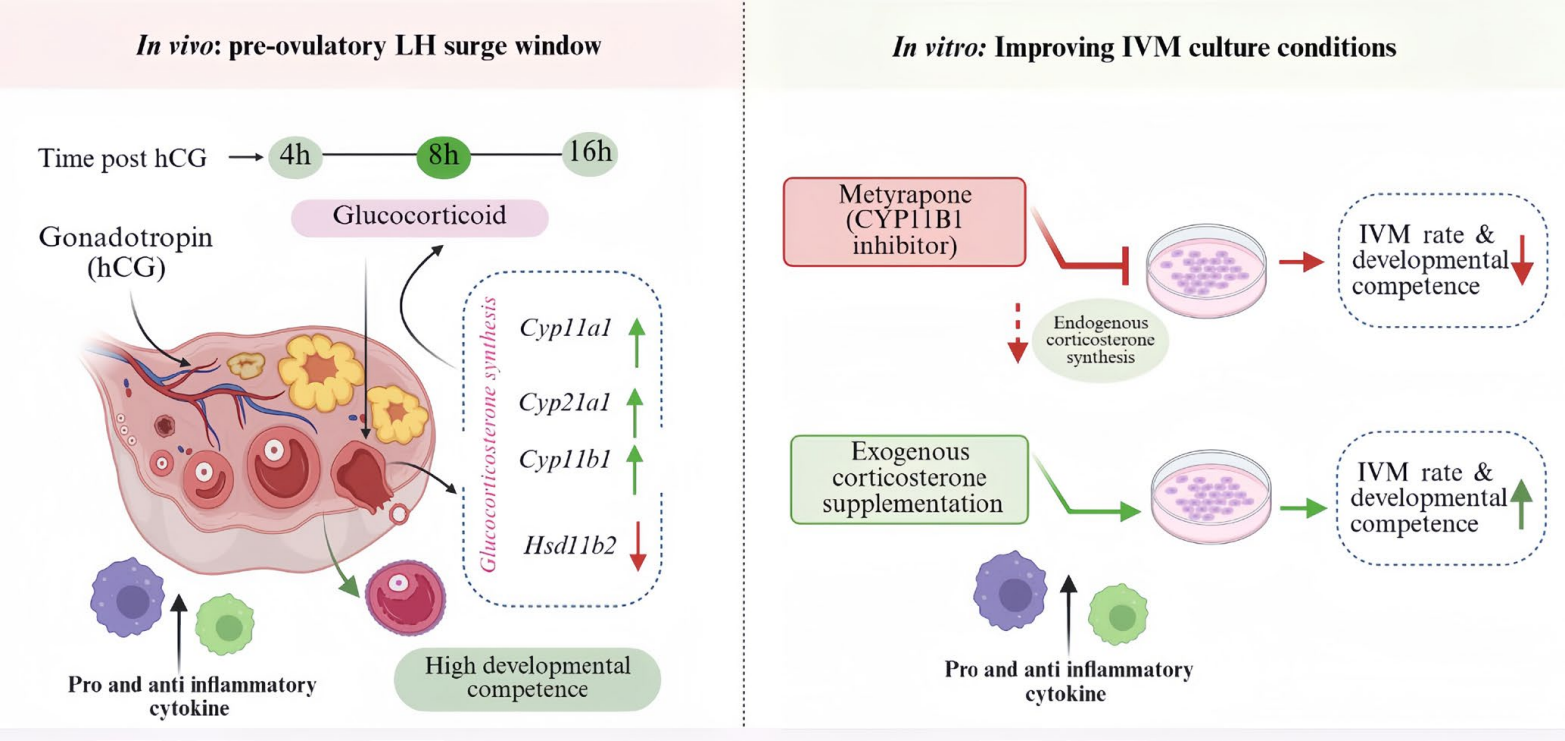
Glucocorticoid is an essential mediator of oocyte developmental competence‐Proposed model. hCG induces a transient increase in follicular corticosterone via upregulation of steroidogenic enzymes and downregulation of *Hsd11b2*, along with increased expression of pro‐ and anti‐inflammatory cytokines and chemokines, supporting oocyte maturation. MET impairs IVM outcomes; however, physiological corticosterone supplementation can improve the IVM rate and developmental competence and mimic in vivo inflammatory cytokine and chemokine expression. This figure was created with BioRender.com.

Taken together, these results indicate that corticosterone is a critical contributor to maintaining an optimal in vitro culture environment that supports oocyte maturation and subsequent embryonic development (Figure 8).

## 4 Discussion

Follicular development and ovulation are precisely regulated complex mechanisms mediated by endocrine and intra‐ovarian signaling networks [31]. This study illustrates that gonadotropin stimulation triggers a temporary, locally controlled glucocorticoid signaling system within the ovary, which may contribute to oocyte maturation and developmental competence, both in vivo and under an IVM environment. A novel finding of this study is the time specificity of the glucocorticoid effect during ovulation. Active glucocorticoid levels and steroidogenic enzyme expression were not consistently elevated during the pre‐ovulatory period; rather, they peaked approximately 8 h post‐hCG stimulation, corresponding with the period of cumulus expansion and cytoplasmic maturation. At this point, pro‐ and anti‐inflammatory cytokines and chemokines' expression were elevated in vivo, suggesting that glucocorticoid activation occurs in parallel with a tightly regulated inflammatory signaling during the pre‐ovulatory period [8, 9, 10, 11, 12, 13, 14, 15, 16, 17, 18, 19, 20, 21, 22, 23, 24, 25, 26, 27, 28, 29, 30, 31, 32, 33]. In several species (human, cattle, horse), follicular cortisol rises around ovulation [34, 35, 36, 37], indicating that a transient glucocorticoid surge may be a conserved feature across mammals. This timing indicates that glucocorticoids are not just by‐products of inflammation but rather function as active regulators of the ovulatory signal, ensuring that inflammatory responses are constructive rather than detrimental.

Our findings further demonstrate that the ovary has a locally regulated glucocorticoid biosynthesis pathway, which is induced by the suppression of *Hsd11b2* and the up‐regulation of *Cyp11a1*, *Cyp21a1*, and especially *Cyp11b1* in response to hCG. In bovine and rat ovaries, CYP11B1 and 11β‐HSD1 have been reported to increase after hCG/LH stimulation, consistent with enhanced local glucocorticoid activation [38, 39, 40]. This synchronized transition toward activation reflects traditional pre‐ovulatory events, wherein cumulus cells and granulosa cells temporarily gain steroidogenic ability in response to LH/hCG. CYP11B1 has traditionally been considered an adrenal‐specific enzyme [41]; nonetheless, its functional activation in cumulus cells and granulosa cells after gonadotropin stimulation indicates that ovarian cells temporarily gain the ability for terminal glucocorticoid production during ovulation. The highest expression of CYP11B1 at 8 h post‐hCG coincides with the period of maximal corticosterone accumulation, underscoring the idea of precisely regulated, stage‐specific steroidogenesis within the follicle.

Previous studies have reported that progesterone is synthesized and secreted during the pre‐ovulatory period [42, 43]. In mice in which the gene encoding the progesterone receptor has been knocked out, ovulation is inhibited [44, 45, 46]. However, when oocytes remaining within the follicles were collected, the surrounding cumulus cell synthesized and secreted hyaluronic acid, which accumulated between the cells and completed cumulus expansion [47, 48, 49]. The oocytes had matured to the MII stage and possessed fertilizing ability. In contrast, studies using RU486, an antagonist of the progesterone receptor, have produced different results. Administration of RU486 to rats inhibited not only ovulation but also the progression of meiotic maturation [50, 51]. Furthermore, during in vitro culture of porcine COCs, the addition of RU486 markedly suppressed cumulus expansion [21]. The apparent discrepancy in these findings can be explained by the fact that RU486 acts not only as an antagonist for the progesterone receptor but also for the glucocorticoid receptor, which is consistent with our findings. Progesterone serves as a precursor for the synthesis of corticosterone during the peri‐ovulatory period. This may explain why GR antagonism (e.g., RU486) can affect cumulus expansion and meiotic progression. The former is essential for follicular rupture, whereas the latter is required for cumulus expansion and oocyte maturation. The observation that CYP11B1 inhibitors suppress both oocyte maturation and cumulus expansion during IVM further supports this hypothesis.

Understanding the role of glucocorticoids in the ovary requires recognizing that its function varies significantly according to the developmental stage. In the follicular growth phase, glucocorticoids induce follicular atresia in the smaller subordinate follicles through inducing apoptosis, thereby assuring the survival of only the dominant follicles [25]. In the dominant follicle, the negative effect is protected by the presence of FSH. In general, FSH enhances progesterone production, a key steroidogenic intermediate within the pathway that may contribute to active glucocorticoid synthesis [52, 53, 54]. However, during the follicular growth phase, FSH enhances the activity of the enzyme HSD11B2. This enzyme transforms active glucocorticoid into an inactive form, thereby preventing potential damage to the follicle. This clarifies why FSH elevates progesterone levels without inducing an increase in active glucocorticoids (corticosterone in rodents) in the dominant follicle during the development phase [25]. However, during the onset of ovulation, the process is reversed. The ovulatory signal initiates the mechanism that produces active glucocorticoids. During this process, progesterone might be converted into an active glucocorticoid, which would subsequently contribute to the maturation and ovulation of the oocytes. This dual role of corticosterone in follicular development and the ovulation process is essential for in vivo follicular development, oocyte maturation, and ovulation. Our study demonstrated that upregulation of *Cyp21a1* and *Cyp11b1*, along with downregulation of *Hsd11b2*, is aligned with increased local glucocorticoid production capacity. However, as other steroids were not measured, changes in pathway flux or redistribution cannot be concluded. Future comprehensive steroid profiling along with glucocorticoid to progesterone ratio measurement is required to clarify the partitioning of progesterone metabolism between reproductive steroidogenesis and glucocorticoid synthesis. Furthermore, while GR expression aligns with the pre‐ovulatory corticosterone surge, its exact signaling role remains unclear due to the lack of functional validation, such as GR antagonism or genetic perturbation. Future studies utilizing cell‐specific knockout models are needed to definitively characterize the GR‐dependent mechanisms in oocyte competence.

In bovine COCs, cortisol is not synthesized de novo; instead, active cortisol is generated locally from cortisone via HSD11B1, and this activation is potentiated by locally produced progesterone through progesterone receptor signaling [55]. Although mice primarily use corticosterone and our study focuses on de novo synthesis (CYP11B1), these findings collectively support the idea that a peri‐ovulatory, locally elevated glucocorticoid milieu within the COC/follicular compartment contributes to oocyte competence. However, in vitro culture system lacks sufficient glucocorticoids as in vivo. Our experiment clearly demonstrated that the inhibition of endogenous corticosterone synthesis by MET led to reduced cumulus expansion, decreased maturation rates, and impaired embryo development, suggesting that *de novo* synthesis of glucocorticoids is essential for oocyte maturation. Indeed, we have used an in vitro pharmacological model, so MET might have potential broader steroidogenic effects that require further investigation under physiological conditions.

Low physiological doses improve cumulus expansion and oocyte maturation rates; conversely, the marked reduction in maturation and blastocyst rates observed at 1000 pg/mL underscores the biphasic nature of glucocorticoid action. While physiological levels are essential, supra‐physiological doses likely over‐suppress the inflammatory environment required for maturation. This excessive GR activation likely shifts the cellular balance toward oxidative stress and apoptosis, similar to pathological responses observed in other cell types [56, 57, 58]. These pathological reactions include disrupting ERK1/2 signaling and compromising the metabolic support that cumulus cells provide to the oocyte [26]. Considering this narrow therapeutic window of IVM, careful concentration optimization is critical to maximize efficacy and prevent dose‐dependent toxic effects. At 8 h post‐hCG, cytokine and chemokine expression remained elevated in vivo, which can be mimicked by supplementing lower doses of glucocorticoids in the maturation medium in vitro. These data suggest that glucocorticoids act through a time‐ and concentration‐specific mechanism with permissive and suppressive effects to maintain balanced inflammatory signaling. This emphasizes the importance of culture systems that include appropriate amounts of glucocorticoids to support oocyte competence and mimic physiological conditions, as endogenous levels are insufficient in vitro.

## 5 Conclusion

This study identified glucocorticoids as critical, locally produced mediators of pre‐ovulatory oocyte maturation after gonadotropin stimulation. Adding physiological levels of corticosterone (100 pg/mL) to the in vitro maturation medium significantly improved meiotic progression, cumulus expansion, embryo development, and mimicked in vivo inflammatory environment. Replicating pre‐ovulatory glucocorticoid signaling in vitro could improve assisted reproductive efficiency in animals and humans.

## Ethics Statement

All procedures during animal experiments were reviewed and approved by the Animal Care and Use Committee of Hiroshima University (Hiroshima, Japan; C23‐36) and conducted according to regulations.

## Conflicts of Interest

Masayuki Shimada is a co‐author of this article and a member of the Editorial Board of the Reproductive Medicine and Biology journal. He was not included in any editorial decision‐making about the acceptance of this paper for publication to reduce bias. Other authors report no conflicts of interest.

## Supporting information


**Figure S1:** Uncropped images of Western blots. CYP11B1 (a), NR3C1 (b), and ACTB (c). The image within the yellow dotted area has been used in Figure 4C. CC: cumulus cell; GC: granulosa cell.
**Figure S2:** Comparative analysis of cytokine and chemokine expression profile of cumulus cells (CC) under in vivo and in vitro culture conditions.
(a) qRT‐PCR analysis of *Il6, Il7, Il‐1β, Cxcl1, Il10* in in vivo derived CC at eCG 48 h and hCG 8 h. Values are presented as the mean ± SEM of more than three independent experiments. Data were analyzed using an unpaired Student's *t*‐test. (b) qRT‐PCR analysis of *Il6, Il7, Il‐1β, Cxcl1, Il10* in 8 h IVM derived CC without corticosterone (0 pg/mL), and with corticosterone (100 pg/mL) and compared with baseline control 0 h (eCG 48 h). Values are presented as the mean ± SEM of more than three independent experiments. Data were analyzed using one‐way ANOVA followed by Tukey HSD post hoc test. Each gene expression was normalized to *Rpl19*. Data were considered statistically significant at *p* < 0.05. Different lowercase letters indicate significant differences (*p* < 0.05). CC, cumulus cell; eCG: equine chorionic gonadotropin; h, Hours; hCG, Human chorionic gonadotropin, IVM, In vitro maturation; SEM, standard error of the mean; ns, non‐significant (*p* > 0.05).

**Table S1:** List of cytokine and chemokine primer sequences used for qRT‐PCR.

## Data Availability

The data that support the findings of this study are available from the corresponding author upon reasonable request.
