# Supplementary material for: Glucocorticoids Improve in Vitro Mouse Oocyte Competence by Mimicking the Physiological Pre‐Ovulatory Environment
Source: Reprod Med Biol. 2026 Apr 2;25(1):e70046. doi: 10.1002/rmb2.70046 (PMC13045403; doi:10.1002/rmb2.70046)
Supplement: Supplementary file 1 — Figure S1: Uncropped images of Western blots. CYP11B1 (a), NR3C1 (b), and ACTB (c). The image within the yellow dotted area has been used in Figure 4C. CC: cumulus cell; GC: granulosa cell. Figure S2: Comparative analysis of cytokine and chemokine expression profile of cumulus cells (CC) under in vivo and in vitro culture conditions. (a) qRT‐PCR analysis of Il6, Il7, Il‐1β, Cxcl1, Il10 in in vivo derived CC at eCG 48 h and hCG 8 h. Values are presented as the mean ± SEM of more than three independent experiments. Data were analyzed using an unpaired Student's t‐test. (b) qRT‐PCR analysis of Il6, Il7, Il‐1β, Cxcl1, Il10 in 8 h IVM derived CC without corticosterone (0 pg/mL), and with corticosterone (100 pg/mL) and compared with baseline control 0 h (eCG 48 h). Values are presented as the mean ± SEM of more than three independent experiments. Data were analyzed using one‐way ANOVA followed by Tukey HSD post hoc test. Each gene expression was normalized to Rpl19. Data were considered statistically significant at p < 0.05. Different lowercase letters indicate significant differences (p < 0.05). CC, cumulus cell; eCG: equine chorionic gonadotropin; h, Hours; hCG, Human chorionic gonadotropin, IVM, In vitro maturation; SEM, standard error of the mean; ns, non‐significant (p > 0.05). [file RMB2-25-e70046-s002.pptx]

## Slide 1
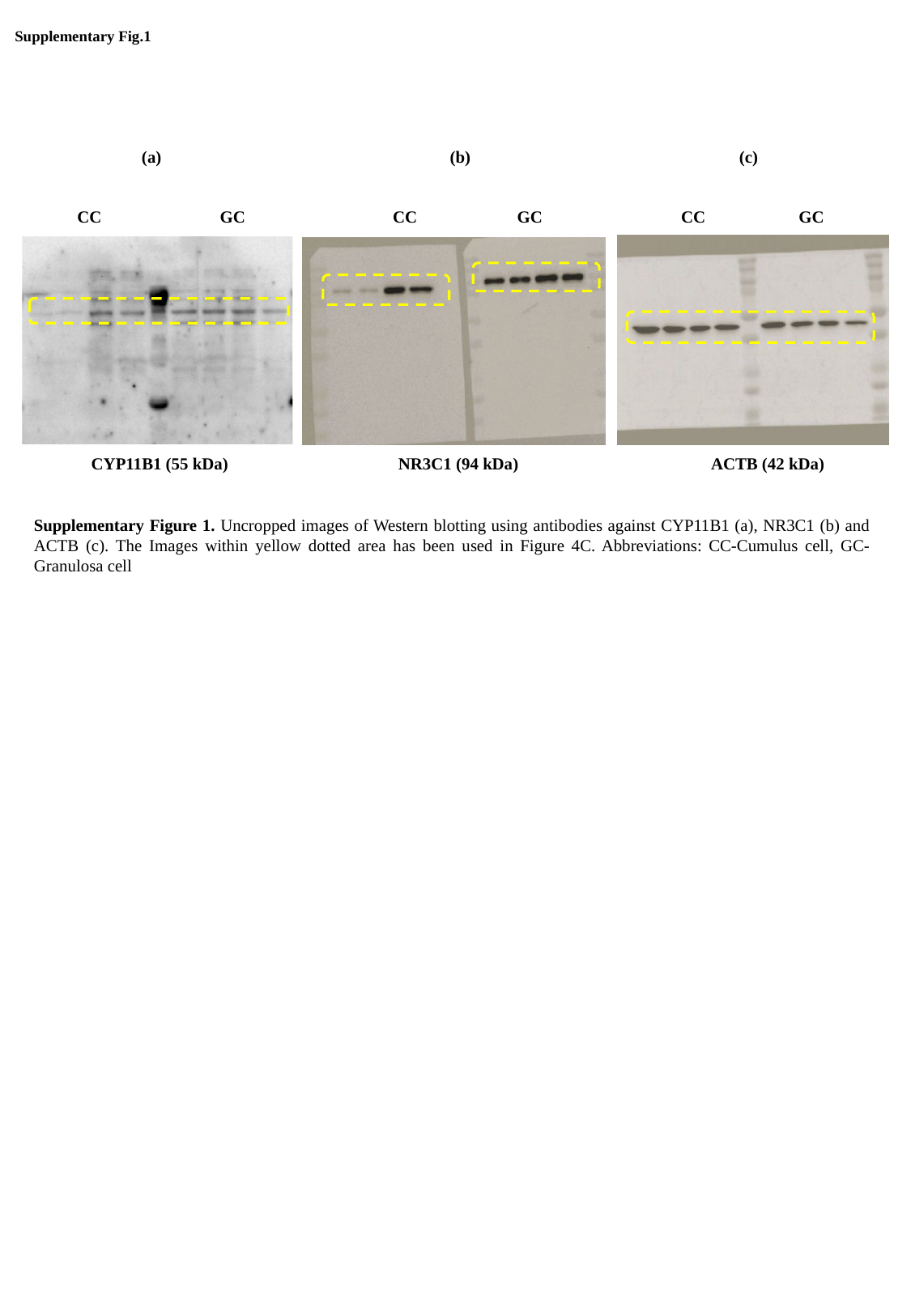

Supplementary Fig.1
(a)
(b)
(c)
CC
GC
CC
GC
CC
GC
CYP11B1 (55 kDa)
NR3C1 (94 kDa)
ACTB (42 kDa)
Supplementary Figure 1. Uncropped images of Western blotting using antibodies against CYP11B1 (a), NR3C1 (b) and ACTB (c). The Images within yellow dotted area has been used in Figure 4C. Abbreviations: CC-Cumulus cell, GC- Granulosa cell

## Slide 2
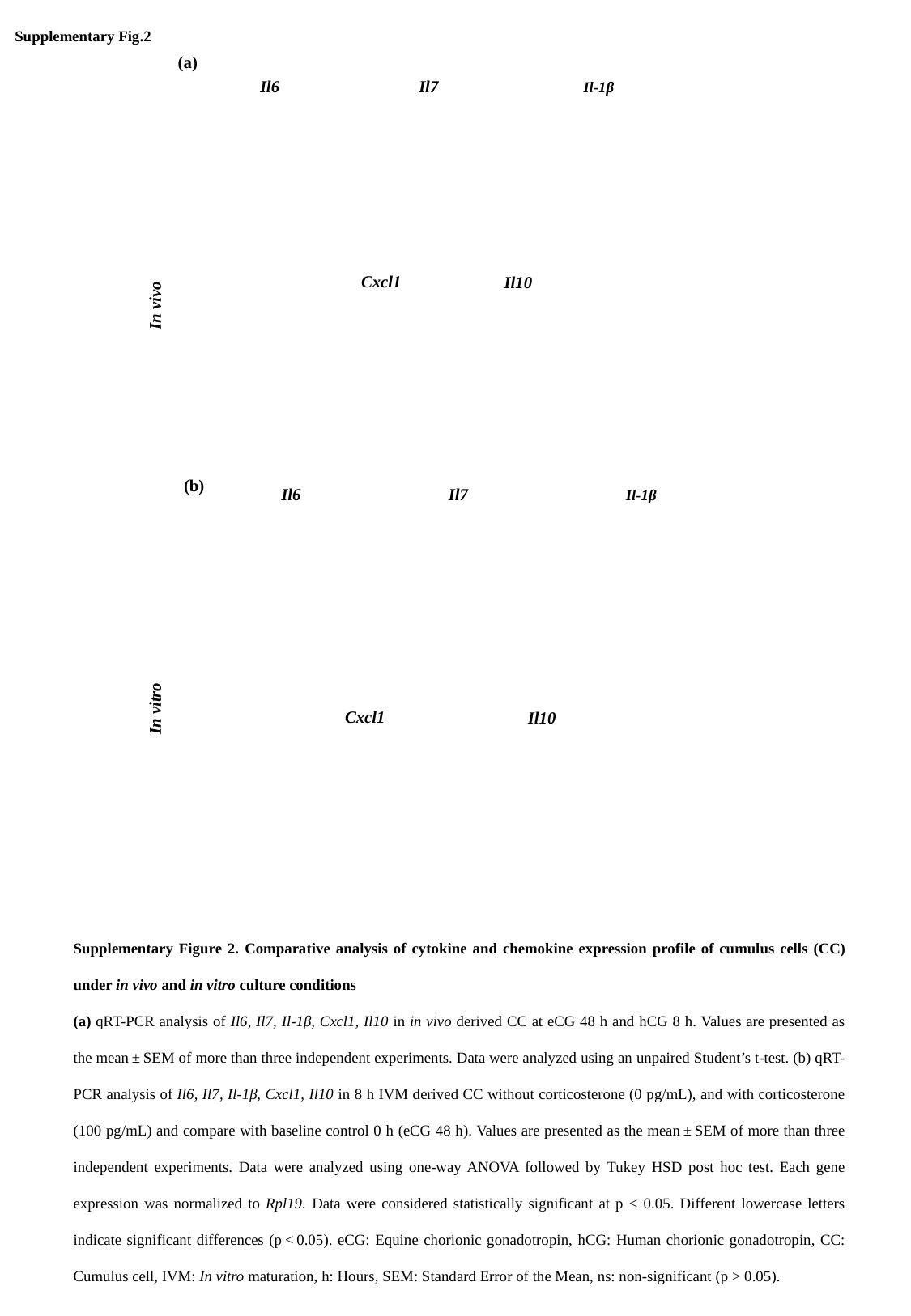

Supplementary Fig.2
(a)
Il6
Il7
Il-1β
Il10
Cxcl1
In vivo
(b)
Il-1β
Il6
Il7
Cxcl1
Il10
In vitro
Supplementary Figure 2. Comparative analysis of cytokine and chemokine expression profile of cumulus cells (CC) under in vivo and in vitro culture conditions
(a) qRT-PCR analysis of Il6, Il7, Il-1β, Cxcl1, Il10 in in vivo derived CC at eCG 48 h and hCG 8 h. Values are presented as the mean ± SEM of more than three independent experiments. Data were analyzed using an unpaired Student’s t-test. (b) qRT-PCR analysis of Il6, Il7, Il-1β, Cxcl1, Il10 in 8 h IVM derived CC without corticosterone (0 pg/mL), and with corticosterone (100 pg/mL) and compare with baseline control 0 h (eCG 48 h). Values are presented as the mean ± SEM of more than three independent experiments. Data were analyzed using one-way ANOVA followed by Tukey HSD post hoc test. Each gene expression was normalized to Rpl19. Data were considered statistically significant at p < 0.05. Different lowercase letters indicate significant differences (p < 0.05). eCG: Equine chorionic gonadotropin, hCG: Human chorionic gonadotropin, CC: Cumulus cell, IVM: In vitro maturation, h: Hours, SEM: Standard Error of the Mean, ns: non-significant (p > 0.05).
