# Supplementary material for: Glucocorticoids Improve in Vitro Mouse Oocyte Competence by Mimicking the Physiological Pre‐Ovulatory Environment
Source: Reprod Med Biol. 2026 Apr 2;25(1):e70046. doi: 10.1002/rmb2.70046 (PMC13045403; doi:10.1002/rmb2.70046)
Supplement: Supplementary file 2 — Table S1: List of cytokine and chemokine primer sequences used for qRT‐PCR. [file RMB2-25-e70046-s001.docx]

**Supplementary Table 1**. List of cytokine and chemokine primer sequences used for qRT-PCR

| **Gene** | **Primer Sequences** | **Product size (bp)** | **Annealing temperature (°C)** | **Accession Number** |
| --- | --- | --- | --- | --- |
| *Il6* | F: 5'-GTCCTTCCTACCCCAATTTCCA-3'  R: 5'-TAACGCACTAGGTTTGCCGA-3' | 154 | 58 | *NM_031168.2* |
| *Il7* | F: 5'-AGTACCTCTTCAATGCAGCCC-3'  R: 5'-TCCTGGGAGCGACTCTTTCA-3' | 129 | 60 | *NM_001313890.1* |
| *Il-1β* | F: 5'-TGCCACCTTTTGACAGTGATG-3'  R: 5'-AAGGTCCACGGGAAAGACAC-3' | 220 | 61 | *NM_008361.4* |
| *Cxcl1* | F: 5'-TGCACCCAAACCGAAGTCAT-3'  R: 5'-CTCCGTTACTTGGGGACACC-3' | 122 | 60 | *NM_008176.3* |
| *Il10* | F: 5'-CGGGAAGACAATAACTGCACCC-3'  R: 5'-CGGTTAGCAGTATGTTGTCCAG-3' | 130 | 60 | *NM_010548.2* |
